# Exploring the construct validity of the Camouflaging Autistic Traits Questionnaire: A factor analytic study

**DOI:** 10.1177/13623613241287964

**Published:** 2024-11-05

**Authors:** Katharine McKinnon, Mackenzie Bougoure, Sici Zhuang, Diana Weiting Tan, Iliana Magiati

**Affiliations:** 1 School of Psychological Science, The University of Western Australia, Australia; 2 School of Education, Macquarie University, Australia

**Keywords:** adults, anxiety, autism spectrum disorders, camouflaging

## Abstract

‘Camouflaging’ is a set of strategies used by autistic people to hide or compensate for their autistic characteristics to fit into predominantly non-autistic social environments. Many researchers have used the Camouflaging Autistic Traits Questionnaire (CAT-Q) to measure camouflaging. However, there have been questions about the construct validity of the CAT-Q as a measurement of camouflaging. The present study examined the extent to which CAT-Q items were distinguishable from other theoretically and empirically related measures of social anxiety, fear of negative evaluation and autistic social traits. Autistic adults (*N* = 308) completed the CAT-Q and social anxiety, fear of negative evaluation and autistic social traits measures. The small-to-large positive associations between camouflaging and social anxiety, fear of negative evaluation and autistic social traits (*r* = 0.14–0.65) were not indicative of collinearity. Three exploratory factor analyses, in which CAT-Q items were factor-analysed together with social anxiety, fear of negative evaluation and autistic social traits items respectively, showed that CAT-Q items generally loaded onto different factors from social anxiety, fear of negative evaluation and autistic social traits items, with the exception of the CAT-Q Assimilation subscale items, which cross-loaded mostly with autistic social traits items. Overall, the CAT-Q items were positively associated with, but also differentiated from, most social anxiety, fear of negative evaluation and autistic social traits items, providing support for the measure’s construct validity.

**Lay abstract:**

Autistic people describe having to mask or ‘camouflage’ their autistic selves to fit into certain social settings. Many researchers have used the CAT-Q to measure the extent to which autistic people engage in camouflaging. However, some researchers have questioned whether the CAT-Q measures camouflaging or whether it measures other related experiences and behaviours associated with social anxiety, fear of being negatively judged or social autistic traits. In our study, we analysed the CAT-Q to check whether it is indeed similar to or different from these related experiences. To do this, we asked 308 autistic adults to complete the CAT-Q and questionnaires about social anxiety, fear of being negatively judged and autistic social features. Then, we put all the CAT-Q items together with the items from each of the other measures in three separate analyses (called factor analyses) to see how the items would group together. These analyses showed us whether camouflaging behaviours are distinguishable and different from, or cluster together with, these other experiences. We found that most of CAT-Q items grouped together separately from the other measures’ items, suggesting that camouflaging differs from these other related experiences. Only some items from one of the CAT-Q subscales clustered together with some social anxiety and autistic items, suggesting these may need to be teased out better in the future. Generally, our findings show that we can use the CAT-Q to measure camouflaging behaviours among autistic people.

Autistic^1^ people commonly experience co-occurring mental health difficulties and are diagnosed with anxiety, social anxiety, depression and other mental health difficulties at rates ranging between 20% and 80%, considerably higher than those reported in the general population (Curnow et al., 2023; Hollocks et al., 2019). Psychosocial experiences contribute to explaining these high rates, as autistic people often encounter more social and communicative challenges than their non-autistic peers, including being misunderstood, stigmatised, discriminated against and excluded (Pearson & Rose, 2021). Resultantly, many autistic people camouflage^2^ their autistic traits to navigate non-autistic social landscapes – a concept called ‘autistic camouflaging’ (hereafter ‘camouflaging’).

Although strategies to monitor and modify social behaviours to fit in and to manage the challenges of the social world are also common in non-autistic people and in other groups (see, for example, Ai et al., 2022, 2024, on camouflaging as impression management and a shared social coping response; see also Hobson & Lee, 2023; van der Putten et al., 2024 on camouflaging in ADHD and developmental language disorder), many autistic adults (e.g. Cage & Troxell-Whitman, 2019), and possibly more autistic women than men (Cook et al., 2021), engage in autistic camouflaging, which involves the conscious and deliberate or unconscious use of several strategies specifically to hide one’s autism and to ‘pass’ as non-autistic, for example, forcing eye contact or preparing social conversation ‘scripts’ (Hull et al., 2017; Libsack et al., 2021). Camouflaging strategies have been described by autistic adolescents and adults as often necessary, adaptive, and helpful for social acceptance, employment, and educational opportunities and as a set of strategies to defend oneself against bullying, discrimination and social isolation (see Zhuang et al., 2023, for review; Lawson, 2020 on ‘adaptive morphing’). Nevertheless, camouflaging has also been described as emotionally painful and exhausting, with more frequent camouflaging use being consistently linked to increased anxiety, depression, suicidality (Beck et al., 2020; Cage & Troxell-Whitman, 2019; Cassidy et al., 2018; Livingston et al., 2019) and autistic burnout (Mantzalas et al., 2022; Raymaker et al., 2020; Zhuang et al., 2023) in cross-sectional studies.

There are different ways to measure autistic people’s camouflaging in research to date. The discrepancy approach measures camouflaging by ‘quantifying differences between internal autistic states reported by autistic people and observable behavioural presentations’ (Cook et al., 2021; p. 7; see also Lai et al., 2017; Williams, 2022), and as such measures the ‘outcomes’ of camouflaging (Williams, 2022) and not the camouflaging behaviours employed or autistic people’s internal processes (see also Cook et al., 2021). Most camouflaging research to date has used self-report checklists (see Hannon et al., 2023 for a review of camouflaging measures). The most commonly used self-report camouflaging scale, the Camouflaging Autistic Traits Questionnaire (CAT-Q; Hull et al., 2019), was developed based on autistic adults’ lived experiences of camouflaging and measures the autistic adults’ self-reported frequency of using different camouflaging behaviours (Hull et al., 2019), rather than the intent, effort or outcomes of camouflaging. An initial exploratory factor analysis indicated that the CAT-Q comprises three subscales (Hull et al., 2019): (a) Masking (efforts to present a non-autistic persona in social situations; e.g. ‘I adjust my body language or facial expressions so that I appear interested by the person I am interacting with’); (b) Assimilation (‘blending-in’ and hiding one’s discomfort in social situations; e.g. ‘In social situations, I feel like I’m “performing” rather than being myself’); and (c) Compensation (behaviours aiming to compensate for autistic social and communication differences; e.g. ‘I have developed a script to follow in social situations’). The CAT-Q has very good to excellent internal consistency, test–retest reliability, and good convergent validity through positive correlations with measures of autistic traits, social and generalised anxiety and depression (Hannon et al., 2023; Hull et al., 2019).

Fombonne (2020) questioned whether measuring camouflaging with the CAT-Q may be confounded by co-occurring social anxiety rather than measuring a distinct set of behaviours associated with camouflaging. Fombonne argued that co-occurring mental health symptoms, such as social anxiety, were not sufficiently explored in camouflaging research and that a demonstration of camouflaging’s construct validity is required. Lai and colleagues (2021) agreed that ‘addressing the unsettled construct validity [of camouflaging] is a priority’ (p. 1037), highlighting a need ‘to identify [camouflaging’s] overlapping factor structures with various current measures and associations with established constructs’ (p. 1037). Similarly, Williams (2022) also urged future research to assess if the CAT-Q items are distinguishable from social anxiety or other mental health or neurodevelopmental features.

In response to these suggestions, the present study examined the extent to which CAT-Q camouflaging items were distinguishable from questionnaire items measuring conceptually and empirically related constructs, namely social anxiety, fear of negative evaluation (FNE) and autistic social traits.

## Social anxiety, fear of negative evaluation, and camouflaging

Rates of clinically elevated social anxiety and/or a diagnosis of social anxiety are common in autistic adults (26%–51%; Hollocks et al., 2019). Social anxiety involves persistent and disabling anxiety around social interactions (McNeil & Randall, 2014) and camouflaging may share at least some conceptual overlap with social anxiety. Like autistic people, socially anxious people typically develop emotional, behavioural and/or social ‘strategies’ to avoid negative evaluation (Wells et al., 1995): these may include intense self-monitoring and a range of safety behaviours to endure or escape social interactions, such as avoidance and social impression management strategies (Ai et al., 2022; Evans et al., 2021; Gray et al., 2019; Hofmann, 2007). Similar strategies have also been described in qualitative studies of autistic camouflaging (Cook et al., 2021; Hull et al., 2017), and some are included in the CAT-Q items. For instance, the CAT-Q Masking subscale has items relating to self-monitoring, while the CAT-Q Assimilation subscale includes items relating to social avoidance.

In the literature to date, positive associations of mostly medium effect size have been reported between the total CAT-Q and social anxiety in autistic adults (*r*s = 0.29 and 0.44 in Hull et al., 2019, 2021, respectively) and camouflaging was a significant predictor of social anxiety (with a small contribution over and above age and autistic traits) in Hull et al. (2021). However, the CAT-Q Assimilation subscale was strongly positively associated with social anxiety (*r*s = 0.60) in Hull et al. (2019). Lei and colleagues (2023) explored the relationship between camouflaging and safety behaviours related to social anxiety in autistic and non-autistic adolescents and found that all three CAT-Q subscales were positively associated with social anxiety (with medium effect size *rs* = 0.25–0.48) after controlling for autistic traits. These consistent associations across studies suggest a relationship between camouflaging and social anxiety, while also suggesting that they are not the same construct based on the effect sizes reported to date.

Fear of negative evaluation (FNE; anxiety over the unfavourable judgement or disapproval of others; Carleton et al., 2007; Lei & Russell, 2021) is a core construct often underlying social anxiety in the general population (Clark & McManus, 2002; Rapee & Heimberg, 1997). Autistic people have reported that camouflaging may be driven by a fear of ostracism and bullying (Cage & Troxell-Whitman, 2019; Hull et al., 2017) and can be a way to avoid being negatively perceived (e.g. Bernardin et al., 2021; Chapman et al., 2022). There is very limited literature on the relationship between FNE and camouflaging, although one study found that FNE was a strong predictor of camouflaging in a cross-sectional path model of sociocultural and individual psychological predictors in autistic adults (Zhuang et al., 2024).

The potential conflation between camouflaging, social anxiety and FNE may not be solely due to similarities between constructs and measurement items, but also because of the high co-occurrence of autism and anxiety, including social anxiety. Twenty to thirty per cent of autistic adults experience clinically elevated social anxiety or a social anxiety disorder diagnosis, and studies employing questionnaires rather than clinical diagnostic interviews suggest rates of clinically elevated social anxiety of more than 50% (Hollocks et al., 2019). It is thus possible that the items developed and included in the CAT-Q based on qualitative descriptions of camouflaging by autistic adults (Hull et al., 2017) may have inadvertently captured autistic adults’ anxiety or FNE relating to social situations and interactions. Given the close intertwining of social anxiety and FNE, it is important to establish that any measure of camouflaging is not conflated with social anxiety or FNE.

## Autistic social traits and camouflaging

Most measures of autistic traits encompass a range of items assessing social interaction and social communication differences and difficulties (hereafter referred to as autistic social traits). Since camouflaging involves hiding or compensating for autistic traits in social situations, it is reasonable to expect a significant positive association between camouflaging and autistic social traits. Indeed, small to medium effect size positive correlations have been reported between autistic social traits and CAT-Q total (*r*s = 0.22–0.33; Hull et al., 2019, 2021). Associations between CAT-Q subscales and autistic traits have varied, such that CAT-Q Masking was not associated with autistic social traits (*r*s = 0.03–0.07), but Compensation was (0.27) and Assimilation showed the strongest associations with BAPQ aloof and pragmatic language subscales at 0.62–0.63 (Hull et al., 2019). However, for the CAT-Q to demonstrate construct validity in measuring camouflaging, CAT-Q items should be distinguishable from the social characteristics commonly associated with autism.

## The present study

Most studies reviewed thus far have examined the correlations between camouflaging and conceptually and empirically similar constructs, including social anxiety and autistic social traits. While these correlational analyses show positive associations between these measures, the construct validity of the CAT-Q has not yet been sufficiently established.

The present study therefore examined the extent to which the CAT-Q items overlap with, or are distinct from, items that measure these related constructs. We first examined the associations between the CAT-Q total and subscale scores and measures of social anxiety, FNE and autistic social traits. Then, we examined the partial relationships between camouflaging and these three variables controlling for social anxiety or autistic social traits respectively, to explore the extent to which these associations were partially or fully explained by the third variable (autistic social traits or social anxiety). Third, we conducted three exploratory factor analyses of the CAT-Q items combined with items from the social anxiety, FNE and autistic social traits’ measures, respectively. This method of combining items from two different measures in a factor analysis is a common approach to evaluate construct validity and has been previously used to investigate measurement and conceptual distinctions between autistic and alexithymic traits (Cuve et al., 2022) and between autistic traits and social anxiety (Lei & Russell, 2021; White et al., 2011).

We hypothesised that (1) CAT-Q will be positively associated with measures of social anxiety, FNE and autistic social traits, but that the correlation coefficients would be smaller than the 0.8 threshold for collinearity (Berry & Feldman, 1985; Vatcheva et al., 2016); and that (2) CAT-Q items will load on different factors from social anxiety, FNE, and autistic social traits’ items.

## Methods

### Participants

Participants were recruited online in 2020–2021 through social media, autism-affiliated organisations and Prolific (https://www.prolific.com).

Individuals who (a) were 18 years or older; (b) had an autism diagnosis or self-identified as autistic;^3^ and (c) could self-report via an online survey in English; were invited to participate. A total of 359 participants responded to our recruitment calls, of whom 51 were excluded due to: (a) not meeting one or more of the inclusion criteria (*n* = 14); (b) not providing consent or completing the survey (*n* = 31); (c) indicating that their data were not valid for use in research (*n* = 1); (d) failing our built-in bot detection for data illegitimacy (*n* = 3); (e) repeating the survey (*n* = 1); and (f) indicating ‘prefer not to say’ for over 30% of their responses (*n* = 1).

Table 1 presents participants’ demographic characteristics (*N* = 308).

**Table 1.** Participants’ socio-demographic characteristics (*N* = 308).

	*N or M*	% or *SD*
Age (years)^a^	36.17	11.62
Diagnosis^b^		
Professional autism diagnosis	265	86
Self-identified as autistic	43	14
Gender		
Female	189	61.4
Male	88	28.6
Non-binary, other genders or undisclosed	31	10.1
Educational level		
Primary school	5	1.6
Secondary school	59	19.2
Trade/technical certificate	43	14
Undergraduate university education	105	34.1
Postgraduate higher education	82	26.6
Other	14	4.5
Ethnicity		
Caucasian	238	77.27
Mixed race	29	9.42
Asian	23	7.47
Hispanic or Latino/Latina	6	1.95
African or Caribbean	5	1.62
Other	7	2.27
Employment status		
Full-time	98	31.82
Part-time	69	22.40
Not working	86	27.92
Student	53	17.21
Prefer not to say	2	0.65
Relationship status		
Single	158	51.30
In a relationship	148	48.05
Prefer not to say	2	0.65
Reported a mental health diagnosis (in past 5 years)		
Anxiety disorders	171	55.52
Depressive disorders	159	51.62
Post-traumatic stress disorder	29	9.42
Obsessive-compulsive disorders	21	6.82
Eating disorders	5	1.62
Other	18	5.84
Number of co-occurring mental health diagnoses reported		
0	95	30.84%
1	71	23.05%
2	102	33.12%
3+	40	12.99%

a Range = 18–76 years.

b There were no differences between diagnosed and self-identified participants in autistic traits reported, and 96.8% of participants had a Broad Autism Phenotype Questionnaire (BAPQ) total score above 3.15, the suggested screening cut-off score for this measure (Hurley et al., 2007).

### Measures

#### Camouflaging

Rated on a 7-point Likert-type scale (from 1 ‘strongly disagree’ to 7 ‘strongly agree’; total score range 25–175; higher scores = more frequent camouflaging), the 25-item Camouflaging Autistic Traits Questionnaire (CAT-Q; Hull et al., 2019) has three subscales (Compensation, Assimilation, and Masking; see Introduction). It has acceptable test–retest reliability after 3 months (*r* = 0.77) and excellent internal consistency (Cronbach’s ⍺ = 0.94). The three-factor model demonstrated a good fit in both autistic and non-autistic samples, and in both men and women (Hull et al., 2019).

#### Autistic traits

Rated on a 6-point Likert-type scale (from 1 ‘very rarely’ to 6 ‘very often’; the total score is averaged and ranges from 1 to 6; higher scores = more autistic traits), the 36-item Broad Autism Phenotype Questionnaire (BAPQ; Hurley et al., 2007) measures autistic traits in the general population, relatives of autistic people, and in autistic people, and has three subscales (aloofness, rigidity, and pragmatic language). The aloofness (12 items) and pragmatic language (12 items) subscales were used in this study to measure autistic social traits. The BAPQ has excellent internal consistency (Cronbach’s ⍺ = 0.91; Hurley et al., 2007). Based on BAPQ cut-off scores by Hurley and colleagues (2007), 91% and 96% of our participants reported BAPQ aloof and pragmatic language subscale scores above cut-off, respectively.

#### Social anxiety

The Social Interaction Anxiety Scale (SIAS-6) and the Social Phobia Scale (SPS-6) are companion short scales (Peters et al., 2012) measuring two core related but distinct elements of social anxiety relating to social interactions and to performance under scrutiny, respectively. Each scale has six items rated on a 5-point Likert-type scale (from 0 ‘not at all characteristic or true of me’ to 4 ‘extremely characteristic or true of me’; scale score range 0–24 and total score range 0–48; higher scores = greater social anxiety). These brief scales have each shown excellent convergent and divergent validity (Peters et al., 2012) and good internal consistency (α = 0.75–0.85) in anxious and non-anxious participants (Le Blanc et al., 2014). The longer 20-item SIAS and SPS scales, from which the short forms were derived, have very good internal consistency and convergent validity with autistic adults (Boulton & Guastella, 2021). Based on the short scales’ development study cut-off scores of 7 for the SIAS-6 and 2 for the SPS-6 with non-autistic participants (Peters et al., 2012), the vast majority of our participants (93%) reported social anxiety above general population cut-offs in these two short scales.

#### Fear of negative evaluation

A brief 8-item version (BFNE-II; Carleton et al., 2006) of the 12-item Brief Fear of Negative Evaluation Scale (BFNES; Leary, 1983) was used. Responses range from 1, ‘not at all characteristic of me’ to 5, ‘extremely characteristic of me’ (total score range 8–40; higher BFNE scores = greater FNE). The 8-item BFNE-II scale had high diagnostic sensitivity and reliability (Cronbach’s ⍺ = 0.95) when completed by autistic adults (Boulton & Guastella, 2021).

### Procedure

Ethics approval was granted by The University of Western Australia (Ref 2021/ET000065). Participants were screened for eligibility before providing written informed consent and completing the questionnaires online on Qualtrics, an online survey platform. The study took about 30–60 min, as participants also completed additional questionnaires as part of a larger study of psychosocial correlates of camouflaging and autistic burnout. Two attention check questions were included and none of our participants failed these. Participants were compensated for their time with the equivalent of AUD20 via direct bank transfer or online gift cards.

### Data analysis plan

Analyses were conducted using IBM SPSS Statistics (Version 26). To examine the first hypothesis, Pearson’s *r* correlations between camouflaging, social anxiety, FNE, and autistic social traits were conducted (*r* = 0.1, 0.3 and 0.5 were considered small, medium or large effect sizes, respectively; Cohen, 1988). In addition, partial correlations between CAT-Q and social anxiety, CAT-Q and FNE (controlling for autistic social traits) and between CAT-Q and autistic social traits (controlling for social anxiety) were calculated.

To examine the second hypothesis, three sets of exploratory factor analyses (EFA) were performed with CAT-Q items entered with either SIAS/SPS, FNE, or autistic social traits items respectively to examine if camouflaging items would load on the same or different factors as social anxiety, FNE and autistic social traits’ items. For the EFAs, maximum likelihood estimation (MLE) was used, as appropriate when factor loadings are unequal and factors are inter-correlated (Field, 2017), which previous research and theory indicated is likely to be the case for all four constructs/measures in this study. Results were interpreted using the pattern matrix, as recommended when there is factor overlap and the solution is rotated (Leandre et al., 2012). Oblique rotation with direct oblimin was used, as correlations between items were expected. A factor loading threshold was set as 0.3, as recommended by Stevens (2002). Scree plot and parallel analysis, as recommended by Costello and Osborne (2005), were used to determine structure solutions which were interpretable, simple and meaningful.

### Community involvement

Some of the authors are neurodivergent and were involved in all aspects of this research including conceptualisation, data analysis and interpretation, and writing up and dissemination. In addition, three paid autistic advisors (who were not authors in this psychometrically focused study on construct validity) provided consultation on the broader research conceptualisation and design, research questions, interpretation and communication of findings of the larger research project within which the current study was embedded.

## Results

### Data screening and descriptive statistics

Skewness and kurtosis values were within recommended limits of 2 and 9, respectively (Field, 2017). Descriptive statistics are presented in Table 2. All total scales and subscales showed good to excellent internal reliability in this study (Cronbach’s α = 0.75–0.95). Our study participants’ total CAT-Q mean score of 129 was similar to others (e.g. 126.7 in Perry et al., 2022; 119.8 in Hull et al., 2019), but somewhat higher than CAT-Q total scores reported in other studies (e.g. 111 in Hull et al., 2021; 106–110 in Oshima et al., 2024; and Tamura et al., 2024 with Japanese autistic adults), possibly due to differences in recruitment, sampling (e.g. Oshima and colleagues excluded self-identifying autistic participants, and both studies included more men than women compared to our sample) and/or sociocultural differences.

**Table 2.** Mean, standard deviation, internal consistency, skewness and kurtosis of main measures (*N* = 308).

	M (or *N*)	SD (or %)	Reliability (Cronbach’s α)	Skewness	Kurtosis
Camouflaging (CAT-Q) Total	129.58	22.94	0.91	–0.77	0.78
CAT-Q Compensation	44.01	11.46	0.88	–0.65	0.03
CAT-Q Masking	40.53	9.55	0.86	–0.96	0.87
CAT-Q Assimilation	45.04	7.51	0.80	–0.79	0.65
Autistic Social Traits (BAPQ^a,b^) Total	4.21	0.65	0.86	–0.17	–0.06
BAPQ Aloof	4.37	0.82	0.87	–0.34	–0.40
BAPQ Pragmatic Language	4.04	0.74	0.78	–0.39	0.31
Social Anxiety (SIAS/SPS total)	26.08	10.59	0.88	–0.15	–0.70
Social Interaction Anxiety Scale (SIAS-6)^b^	14.00	4.93	0.75	–0.36	0.03
Social Phobia Scale (SPS-6)^b^	12.08	6.88	0.89	–0.07	–1.02
Brief Fear of Negative Evaluation (BFNE) total	30.10	8.84	0.95	–0.67	–0.62

*SD*: standard deviation; CAT-Q: Camouflaging Autistic Traits Questionnaire; BAPQ: Broad Autism Phenotype Questionnaire; SPS: Social Phobia Scale; BFNE: Brief Fear of Negative Evaluation.

a Only the ‘Pragmatic Language’ and ‘Aloof’ BAPQ subscale total raw scores were included from the BAPQ in this study.

b For the BAPQ, the suggested cut off for the aloof subscale is 3.25 and for the pragmatic language 2.75 (Hurley et al., 2007); for the SIAS the suggested clinically elevated cut-off of the short forms for the general population is 7 and for the SPS it is 2 (Peters et al., 2012); overall most of our participants (91% for aloof subscale and 96% for pragmatic language subscale) reported autistic social traits and social anxiety (93%) exceeding the suggested cut-off scores.

Table 3 presents inter-correlations between age, gender, diagnostic status (professionally diagnosed vs self-identified) and CAT-Q, SIAS/SPS, BFNE and BAPQ. These were all either non-significant or of small effect sizes. The main analyses were therefore carried out with the whole sample.

**Table 3.** Correlations between camouflaging (CAT-Q), autistic social traits (BAPQ^a^), social anxiety (SIAS/SPS), fear of negative evaluation (BFNE), age, gender and diagnostic status (professionally diagnosed/self-identified) [and partial correlations between CAT-Q, BAPQ^a^, SIAS/SPS and BFNE]^#^.

	CATQ Total	CATQ Comp	CATQ Masking	CATQ Assim	BAPQ Prag + Aloof^b^	BAPQ Pragmatic Language	BAPQ Aloof	SIAS/ SPS	BFNE	Age	Gender
CATQ total											
CATQ Comp	0.87***										
CATQ Masking	0.84***	0.61***									
CATQ Assim	0.66***	0.37***	0.36***								
BAPQ^a^ Prag + Aloof	0.33***	0.24***	0.00	0.66***							
	*[0.09]* ^#^	*[0.05]* ^#^	[-0.18**] ^#^	*[0* . 49***]^#^							
BAPQ Prag	0.27***	0.27***	-0.02	0.42***	0.81***						
BAPQ Aloof	0.29***	0.14*	0.02	0.65***	0.85***	0.38***					
SIAS/SPS	0.45***	0.34***	0.25***	0.54***	0.60***	0.56***	0.44***				
	*[0* . 33***]^#^	[0.25***] ^#^	*[0* . 30***]^#^	[0.25***] ^#^							
BFNE	0.40***	0.28***	0.38***	0.29**	0.15**	0.26***	0.01	0.55***			
	[0.37***] ^#^	[0.26***] ^#^	[0.38***] ^#^	[0.25***] ^#^							
Age	-0.02	-0.09	-0.05	0.14*	0.12*	-0.02	0.22***	-0.14*	-0.29***		
Gender	0.24***	0.23***	0.22***	0.11	0.02	0.11	-0.06	0.08	0.11	-0.01	
Diagnostic status	0.04	-0.01	0.05	0.08	0.05	-0.02	0.10	-0.01	-0.01	0.10	0.10

*Note*. CAT-Q Com = CAT-Q Compensation; CAT-Q Assim = CAT-Q Assimilation; BAPQ Prag + Aloof = BAPQ Pragmatic Language’ and ‘Aloof’ subscale; SIAS/SPS: Social Interaction Anxiety Scale/Social Phobia Scale; BFNE: Brief Fear of Negative Evaluation; Diagnostic status = professionally diagnosed or self-identifying as autistic.

# *[partial correlations]*: CAT-Q with BAPQ^a^ controlling for social anxiety (SIAS/SPS); CAT-Q with social anxiety (SIAS/SPS) and FNE controlling for autistic social traits. In bold, correlations >0.3 and *p* < 0.001. ^a^Only the ‘Pragmatic Language’ and ‘Aloof’ BAPQ subscale total raw scores were included from the BAPQ in this study.

* *p* < 0.05, ** *p* < 0.01, *** *p* < 0.001.

### Associations between camouflaging, social anxiety, FNE and autistic social traits (Hypothesis 1)

Consistent with the prediction that the CAT-Q would show positive, but not larger than 0.8, correlations with measures of social anxiety, FNE and autistic social traits, correlations between the CAT-Q total or subscale scores and SIAS/SPS, BFNE and BAPQ were all <0.66 (see Table 3), with the largest associations between CAT-Q Assimilation and BAPQ Aloof + Pragmatic Language autistic social traits (0.66).

Partial correlations between camouflaging (CAT-Q) total and subscales and social anxiety controlling for autistic social traits ranged from 0.25 to 0.30 (Table 3), and partial correlations between autistic social traits and camouflaging controlling for social anxiety ranged from −0.18 to 0.49 (Table 3). Overall, the partial associations were mostly smaller in effect size than the Pearson’s *r* correlations, although still significant and of medium effect size, suggesting that to some extent the relationship between the variables was partially, but not fully, accounted for by the third variable. Only the relationship between CAT-Q Compensation and autistic social traits was no longer significant (partial *r* = 0.05) when social anxiety was partialled out, suggesting that this relationship was fully accounted for by social anxiety. The relationships between FNE and CAT-Q remained largely the same even after partialling out autistic social traits.

### Examining construct overlap between camouflaging and social anxiety, FNE and autistic social traits’ items (Hypothesis 2)

The Kaiser-Meyer-Olkin (KMO) factorability assumptions’ values were 0.90 for social anxiety, 0.91 for FNE and 0.89 for autistic social traits, above the minimum of 0.70 recommended by Kaiser (1974), indicating that the data were suitable for factor analysis.

Over all three FAs conducted, CAT-Q items generally showed little cross-loading with other non-camouflaging factors: one CAT-Q Masking item loaded on two factors with non-camouflaging items (‘I always think about the impression I make on other people’ cross-loaded into a social anxiety factor and a social autistic traits’ factor; see Tables 4 and 5); and one CAT-Q-Assimilation item (‘When talking to other people, I feel like the conversation flows naturally’) cross-loaded into the social autistic traits’ factor; see Table 6). In all other instances of cross-loading (see Table 4, Table 5 and Table 6), some of the CAT-Q items may have on some occasions cross-loaded with another CAT-Q factor, but not with social anxiety, FNE or social autistic traits’ factors.

**Table 4.** Factor loadings of exploratory factor analysis of the Camouflaging Autistic Traits Questionnaire (CAT-Q) and the Social Interaction Anxiety Scale/Social Phobia Scale (SIAS/SPS) items (*N* = 308).

Scale/item		Factor loadings			
		1	2	3	4
CAT-Q_M1	I monitor my body language or facial expressions so that I appear relaxed.	0.79			
CAT-Q_M2	I adjust my body language or facial expressions so that I appear interested by the person I am interacting with.	0.74			
CAT-Q_M3	*I always think about the impression I make on other people*	*0.39*	*0.33*		
CAT-Q_M4	I don’t feel the need to make eye contact with other people if I don’t want to. (R)	0.40			
CAT-Q_M5	I monitor my body language or facial expressions so that I appear interested by the person I am interacting with.	0.72			
CAT-Q_M6	I am always aware of the impression I make on other people.	0.33			
CAT-Q_M7	I adjust my body language or facial expressions so that I appear relaxed.	0.70			
CAT-Q_M8	In social interactions, I do not pay attention to what my face or body are doing. (R)	0.66			
CAT-Q_A1	*I rarely feel the need to put on an act in order to get through a social situation. (R)*	*0.33*			*0.35*
CAT-Q_A2	*In social situations, I feel like I’m ‘performing’ rather than being myself*.	*0.38*			*0.44*
CAT-Q_A3	I need the support of other people in order to socialise.				0.36
CAT-Q_A4	I have to force myself to interact with people when I am in social situations.				0.66
CAT-Q_A5	When in social situations, I try to find ways to avoid interacting with others.				0.54
CAT-Q_A6	I feel free to be myself when I am with other people. (R)				0.64
CAT-Q_A7	When talking to other people, I feel like the conversation flows naturally. (R)				0.67
CAT-Q_A8	In social situations, I feel like I am pretending to be ‘normal’.				0.59
CAT-Q_C1	*When I am interacting with someone, I deliberately copy their body language or facial expressions*.	*0.31*		*-0.36*	
CAT-Q_C2	I have developed a script to follow in social situations (for example, a list of questions or topics of conversation).			-0.49	
CAT-Q_C3	I will repeat phrases that I have heard others say in the exact same way that I first heard them.			-0.47	
CAT-Q_C4	In my own social interactions, I use behaviours that I have learned from watching other people interacting.			-0.66	
CAT-Q_C5	I practice my facial expressions and body language to make sure they look natural.			-0.48	
CAT-Q_C6	I have tried to improve my understanding of social skills by watching other people.			-0.64	
CAT-Q_C7	I have researched the rules of social interactions (for example, by studying psychology or reading books on human behaviour) to improve my own social skills.			-0.51	
CAT-Q_C8	I learn how people use their bodies and faces to interact by watching television or films, or by reading fiction.			-0.91	
CAT-Q_C9	I have spent time learning social skills from television shows and films, and try to use these in my interactions.			-0.97	
SIAS1	I have difficulty making eye contact with others.		0.35		
SIAS2	I find it difficult mixing comfortably with the people I work with.				0.48
SIAS3	I tense up if I meet an acquaintance on the street.				0.54
SIAS4	I feel tense if I am alone with just one person.		0.30		
SIAS5	I have difficulty talking with other people.				0.53
SIAS6	I find it difficult to disagree with another’s point of view.				
SPS7	I get nervous that people are staring at me as I walk down the street.		0.77		
SPS8	I worry about shaking or trembling when I’m watched by other people.		0.54		
SPS9	I would get tense if I had to sit facing other people on a bus or train.		0.75		
SPS10	I worry I might do something to attract the attention of other people.		0.76		
SPS11	When in an elevator, I am tense if people look at me.		0.86		
SPS12	I can feel conspicuous standing in a line.		0.77		
					

*Note*. CAT-Q_M: CAT-Q Masking; CAT-Q_A: CAT-Q Assimilation; CAT-Q_C: CAT-Q Compensation; SIAS/SPS Social Interaction Anxiety Scale/Social Phobia Scale. Reverse-scored items denoted with (R). Items in *italics* are cross-loaded.

**Table 5.** Factor loadings of exploratory factor analysis of the Camouflaging Autistic Traits Questionnaire (CAT-Q) and the Brief Fear of Negative Evaluation Scale (BFNE) items (*N* = 308).

Scale/item		Factor loadings			
		1	2	3	4
CAT-Q_M1	I monitor my body language or facial expressions so that I appear relaxed.	0.83			
CAT-Q_M2	I adjust my body language or facial expressions so that I appear interested by the person I am interacting with.	0.76			
CAT-Q_M3	*I always think about the impression I make on other people*	*0.32*	*-0.43*		
CAT-Q_M4	I don’t feel the need to make eye contact with other people if I don’t want to. (R)	0.37			
CAT-Q_M5	I monitor my body language or facial expressions so that I appear interested by the person I am interacting with.	0.73			
CAT-Q_M6	I am always aware of the impression I make on other people.	0.31			
CAT-Q_M7	I adjust my body language or facial expressions so that I appear relaxed.	0.73			
CAT-Q_M8	In social interactions, I do not pay attention to what my face or body are doing. (R)	0.68			
CAT-Q_A1	*I rarely feel the need to put on an act in order to get through a social situation. (R)*	*0.32*			*0.35*
CAT-Q_A2	*In social situations, I feel like I’m ‘performing’ rather than being myself*.	*0.33*			*0.50*
CAT-Q_A3	I need the support of other people in order to socialise.				0.39
CAT-Q_A4	I have to force myself to interact with people when I am in social situations.				0.69
CAT-Q_A5	When in social situations, I try to find ways to avoid interacting with others.				0.60
CAT-Q_A6	I feel free to be myself when I am with other people. (R)				0.65
CAT-Q_A7	When talking to other people, I feel like the conversation flows naturally. (R)				0.61
CAT-Q_A8	In social situations, I feel like I am pretending to be ‘normal’.				0.60
CAT-Q_C1	When I am interacting with someone, I deliberately copy their body language or facial expressions.			0.36	
CAT-Q_C2	I have developed a script to follow in social situations (for example, a list of questions or topics of conversation).			0.46	
CAT-Q_C3	I will repeat phrases that I have heard others say in the exact same way that I first heard them.			0.47	
CAT-Q_C4	In my own social interactions, I use behaviours that I have learned from watching other people interacting.			0.65	
CAT-Q_C5	I practice my facial expressions and body language to make sure they look natural.			0.48	
CAT-Q_C6	I have tried to improve my understanding of social skills by watching other people.			0.63	
CAT-Q_C7	I have researched the rules of social interactions (for example, by studying psychology or reading books on human behaviour) to improve my own social skills.			0.50	
CAT-Q_C8	I learn how people use their bodies and faces to interact by watching television or films, or by reading fiction.			0.91	
CAT-Q_C9	I have spent time learning social skills from television shows and films, and try to use these in my interactions.			0.96	
BFNE1	I worry about what other people will think of me even when I know it doesn’t make any difference.		-0.89		
BFNE2	I am frequently afraid of other people noticing my shortcomings.		-0.83		
BFNE3	I am afraid that others will not approve of me.		-0.89		
BFNE4	I am afraid that people will find fault with me.		-0.88		
BFNE5	When I am talking to someone, I worry about what they may be thinking about me.		-0.86		
BFNE6	I am usually worried about what kind of impression I make.		-0.86		
BFNE7	If I know someone is judging me, it tends to bother me.		-0.80		
BFNE8	I often worry that I will say or do the wrong things.		-0.73		

*Note.* CAT-Q_M: Masking; CAT-Q_A Assimilation; CAT-Q_C Compensation; BFNE: Brief Fear of Negative Evaluation scale. Reverse-scored items denoted with (R). Items in italics are cross-loaded.

**Table 6.** Factor loadings of exploratory factor analysis of the Camouflaging Autistic Traits Questionnaire (CAT-Q) and the Broad Autism Phenotype Questionnaire (BAPQ) autistic social traits’ items (*N* = 308).

Scale/item		Factor loadings			
		1	2	3	4
CAT-Q_M1	I monitor my body language or facial expressions so that I appear relaxed.			-0.74	
CAT-Q_M2	I adjust my body language or facial expressions so that I appear interested by the person I am interacting with.			-0.70	
CAT-Q_M3	I always think about the impression I make on other people			-0.40	
CAT-Q_M4	I don’t feel the need to make eye contact with other people if I don’t want to. (R)			-0.38	
CAT-Q_M5	*I monitor my body language or facial expressions so that I appear interested by the person I am interacting with*.	*0.31*		*-0.69*	
CAT-Q_M6	I am always aware of the impression I make on other people.			-0.32	
CAT-Q_M7	*I adjust my body language or facial expressions so that I appear relaxed*.	*0.31*		*-0.64*	
CAT-Q_M8	In social interactions, I do not pay attention to what my face or body are doing. (R)			-0.63	
CAT-Q_A1	I rarely feel the need to put on an act in order to get through a social situation. (R)			-0.39	
CAT-Q_A2	In social situations, I feel like I’m ‘performing’ rather than being myself.			-0.48	
CAT-Q_A3	I need the support of other people in order to socialise.				
CAT-Q_A4	I have to force myself to interact with people when I am in social situations.		0.57		
CAT-Q_A5	When in social situations, I try to find ways to avoid interacting with others.		0.70		
CAT-Q_A6	*I feel free to be myself when I am with other people. (R)*		*0.44*	*-0.38*	
CAT-Q_A7	*When talking to other people, I feel like the conversation flows naturally. (R)*		*0.48*		*0.36*
CAT-Q_A8	*In social situations, I feel like I am pretending to be ‘normal’*.		*0.31*	*-0.38*	
CAT-Q_C1	*When I am interacting with someone, I deliberately copy their body language or facial expressions*.	*0.39*		*-0.34*	
CAT-Q_C2	I have developed a script to follow in social situations (for example, a list of questions or topics of conversation).	0.44			
CAT-Q_C3	I will repeat phrases that I have heard others say in the exact same way that I first heard them.	0.41			
CAT-Q_C4	In my own social interactions, I use behaviours that I have learned from watching other people interacting.	0.64			
CAT-Q_C5	I practice my facial expressions and body language to make sure they look natural.	0.53			
CAT-Q_C6	I have tried to improve my understanding of social skills by watching other people.	0.62			
CAT-Q_C7	I have researched the rules of social interactions (for example, by studying psychology or reading books on human behaviour) to improve my own social skills.	0.49			
CAT-Q_C8	I learn how people use their bodies and faces to interact by watching television or films, or by reading fiction.	0.93			
CAT-Q_C9	I have spent time learning social skills from television shows and films, and try to use these in my interactions.	0.94			
BAPQ_A1 (R)	I like being around other people. (R)		0.82		
BAPQ_A2	I would rather talk to people to get information than socialise.		0.54		
BAPQ_A3 (R)	I enjoy being in social situations. (R)		0.89		
BAPQ_A4 (R)	People find it easy to approach me. (R)		0.38		
BAPQ_A5 (R)	I look forward to situations where I can meet new people. (R)		0.71		
BAPQ_A6	*When I make conversation it is just to be polite*.		*0.36*	*-0.31*	
BAPQ_A7 (R)	I am good at making small talk. (R)		0.43		
BAPQ_A8 (R)	I feel like I am really connecting with other people. (R)		0.65		
BAPQ_A9	Conversation bores me.		0.53		
BAPQ_A10	*I am warm and friendly in my interactions with others. (R)*		*0.47*	*0.41*	
BAPQ_A11	I prefer to be alone rather than with others.		0.68		
BAPQ_A12	I enjoy chatting with people. (R)		0.86		
BAPQ_PL1	I find it hard to get my words out smoothly.				0.43
BAPQ_PL2	It’s hard for me to avoid getting sidetracked in a conversation.				0.61
BAPQ_PL3	*I am ‘in-tune’ with the other person during conversation. (R)*		*0.39*	*0.31*	*0.33*
BAPQ_PL4	My voice has a flat or monotone sound to it.				
BAPQ_PL5	*I feel disconnected or ‘out of sync’ in conversations with others*.		*0.35*		*0.42*
BAPQ_PL6	People ask me to repeat things I’ve said because they don’t understand.				0.43
BAPQ_PL7	I have been told that I talk too much about certain topics.				0.41
BAPQ_PL8	I speak too loudly or softly.				0.51
BAPQ_PL9	*I can tell when someone is not interested in what I am saying. (R)*			*0.31*	*0.33*
BAPQ_PL10	I leave long pauses in conversation.				
BAPQ_PL11	I lose track of my original point when talking to people.				0.62
BAPQ_PL12	I can tell when it is time to change topics in conversation. (R)				0.53

*Note*. CAT-Q_M Masking; CAT-Q_A Assimilation; CAT-Q_C Compensation; BAPQ-A Aloof; BAPQ-PL pragmatic language. Reverse-scored items denoted with (R). Items in *italics* are cross-loaded.

#### Camouflaging and social anxiety items

**Figure 1.**
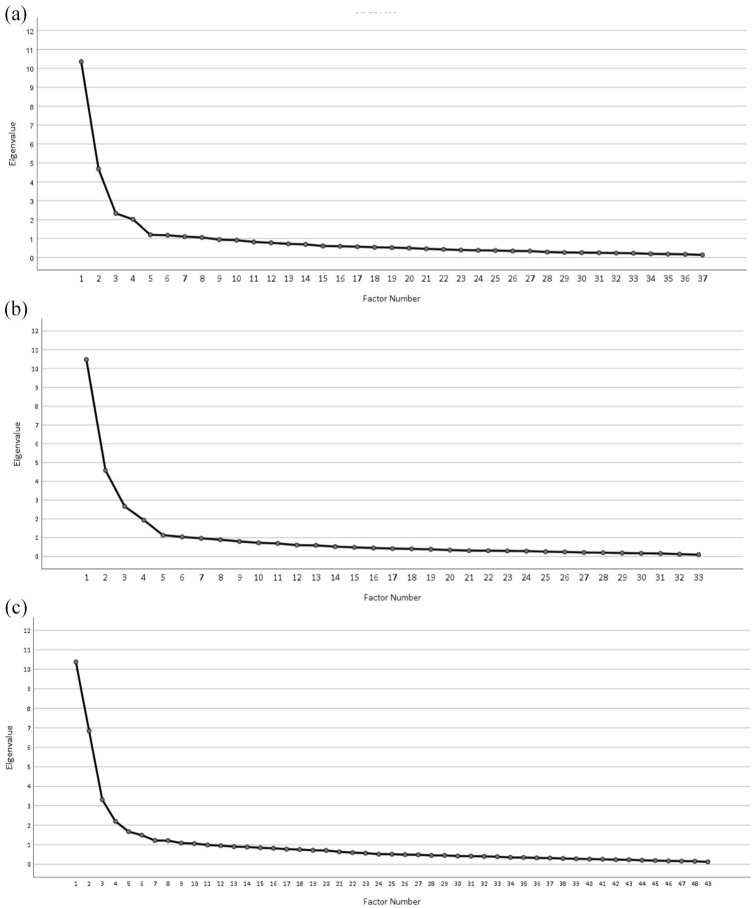
Scree plot of the three factor analyses conducted with camouflaging (CAT-Q) and social anxiety (SIAS/SPS), fear of negative evaluation (BFNE) and autistic social traits (BAPQ) respectively: (a) scree plot for CAT-Q and SIAS/SPS Items, (b) scree plot for CAT-Q and BFNE Items and (c) scree plot for CAT-Q and BAPQ Items.

Parallel analysis indicated up to eight factors could be extracted. However, on the basis of the scree plot (Figure 1(a)), four factors were retained explaining a total of 46.9% of the variance, as this provided an interpretable, simple and meaningful factor solution (Table 4). All CAT-Q items met the factor loading threshold of 0.3, and only one social anxiety item fell below this threshold. In this and the other two EFAs, these cross-loadings were not addressed and all items were retained, as the aim of the EFAs was not to extract factors, as in standard factor analysis, but rather to examine the extent to which items loaded into separable and meaningful factors.

Factor 1 contained CAT-Q items only (all eight CAT-Q Masking, along with two Assimilation and one Compensation items) and explained 26.0% of the variance. Factor 2 mostly contained social anxiety items (8 of the 12 SPS/SIAS items) and one CAT-Q Masking item, explaining an additional 11.5% of the variance. Factor 3 contained only CAT-Q Compensation items, with all nine Compensation items loading on this factor explaining another 4.2% of the total variance. Finally, Factor 4 contained mostly CAT-Q Assimilation items (all eight CAT-Q Assimilation items loaded onto this factor) and three social anxiety items, explaining 4.9% of the variance.

These results supported Hypothesis 2, as CAT-Q and social anxiety items largely loaded onto different factors.

#### Camouflaging and FNE items

Parallel analysis indicated up to eight factors could be extracted. Based on the scree plot (see Figure 1(b)), four factors explaining a total of 54.2% of the variance were retained, as this provided the most interpretable and parsimonious solution. All CAT-Q and BFNE items met the factor loading threshold of 0.3. There was a small amount of cross-loading, as only three CAT-Q items loaded on two factors (see Table 5).

Factor 1 (29.8% of the variance) contained only CAT-Q items (all eight CAT-Q Masking and two Assimilation items). The second factor (13.5% of variance) contained mostly FNE items (all eight BFNE items and one CAT-Q Masking item). Factor 3 (4.5% of variance) comprised all nine CAT-Q Compensation items and factor four (6.5% of variance) contained all eight CAT-Q Assimilation items. These findings supported Hypothesis 2, as CAT-Q and BFNE items largely loaded onto different factors.

#### Camouflaging and autistic social traits’ items

Parallel analysis indicated up to seven factors could be extracted, but based on the scree plot and the most interpretable and parsimonious solution (Figure 1(c)), four factors were retained (Table 6), explaining a total of 41.7% of the variance. Three items did not meet the factor loading threshold of 0.3 (one CAT-Q Assimilation and two BAPQ Pragmatic subscale items), which were retained.

Compared to the previous two factor analyses, a larger number of cross-loading items was found (10 of the 49 items). Factor 1 (19.5% of variance) contained only CAT-Q items (all nine CAT-Q Compensation and two CAT-Q Masking items). Factor 2 (13.3% of variance) contained 14 BAPQ items (12 from the Aloof and two from the Pragmatic Language subscales) and five of the eight CAT-Q Assimilation items. Factor 3 (4.7% of variance) contained 13 CAT-Q items (all eight CAT-Q Masking, 4 Assimilation and one Compensation items) and four BAPQ Aloof/Pragmatic Language items. Finally, Factor 4 (4.4% of variance) contained 10 social autistic trait items (10 of the 12 BAPQ Pragmatic Language items) and one CAT-Q Assimilation item.

These results only partially supported Hypothesis 2. Although many camouflaging and social autistic trait items loaded onto different factors, the CAT-Q Assimilation subscale items in particular appeared to be less distinct, as over half of the CAT-Q Assimilation items loaded onto Factor 2, which was otherwise a factor containing mostly BAPQ items.

## Discussion

The present study explored the construct validity of the CAT-Q by examining the degree to which CAT-Q items were related to and/or distinct from items measuring social anxiety, FNE, and autistic social traits. Our findings indicated that the CAT-Q was significantly positively correlated with the other measures, but the strength of the associations did not indicate collinearity. Furthermore, our factor analyses showed that most CAT-Q items loaded largely separately from social anxiety, FNE, and autistic social traits’ items, with the exception of CAT-Q Assimilation subscale items, some of which cross-loaded mostly with social autistic trait items. These findings overall provide support for the CAT-Q’s construct validity, with some caveats that are discussed below.

The first hypothesis that the CAT-Q and measures of social anxiety, FNE and autistic social traits would be positively related, but smaller than 0.8, was supported, as all inter-correlations were <0.66. Hull et al. (2019, 2021) and Lei et al. (2023) also found positive small-to-large effect size CAT-Q associations with autistic traits and social anxiety. The small-medium associations between the CAT-Q Masking and Compensation subscales (*r*s = 0.00–0.38) strongly point towards no or a weak relationship between these CAT-Q subscales and social anxiety, FNE and autistic social traits, however the medium-to-large associations between the CAT-Q Assimilation subscale and the other measures examined in this study (0.29–0.66) suggest a stronger relationship between this particular CAT-Q subscale and related social anxiety and autistic experiences and characteristics. The relationships between the CAT-Q subscales and BFNE and social anxiety respectively appeared to be generally similar in terms of the associations’ effect sizes (mostly medium; see Table 3); we note, however, that the CAT-Q Assimilation subscale was more strongly correlated with social anxiety (0.54) as compared to FNE (0.29), whereas the association between CAT-Q Masking and FNE was somewhat larger (0.38) compared to its relationship with social anxiety (0.25). Tentatively, it is possible that there are differential relationships between different aspects of camouflaging, FNE and social anxiety which require further research. If replicated in future studies, such differential associations could possibly be explained by the focus of the CAT-Q Assimilation items on avoidance and ‘putting on a act’ to hide one’s discomfort and anxiety in social interactions (which relate more to social anxiety), as compared to the CAT-Q Masking items, which describe strategies to manage others’ impressions (which likely relate more closely to, and could be driven by, FNE).

In the exploratory partial correlations, most of the relationships between CAT-Q, social anxiety and autistic social traits remained significant, but were smaller than the zero-order correlations when partialling out the effects of the other variable, suggesting direct but small/medium effect size relationships. Only the relationship between CAT-Q Compensation and BAPQ autistic social traits was fully accounted for by social anxiety.

The factor analyses we conducted, entering CAT-Q with the other measures’ items together, showed that the CAT-Q items were mostly separate from the social anxiety items, as they largely loaded onto different factors. The CAT-Q items also mostly loaded onto different factors from FNE items, indicating that they were also separable factorially from FNE items. These findings support the construct validity of the CAT-Q based on few to no cross-loadings with, and items largely separable from, social anxiety and FNE items. It is also possible that the differentiation between items in this study’s factor analyses could reflect the different focus of CAT-Q and social anxiety and FNE items in the measures employed: the CAT-Q items largely reflect frequency of *behaviours*, while the social anxiety and FNE measures used tend to include more items focusing on anxious *thoughts/cognitions and emotions*, therefore it is possible that the different factor loadings may to some extent also reflect the distinction between mostly behaviourally focused versus mostly cognitively or emotionally focused experiences. In support of this possibility, Lei et al. (2023) found construct overlap between the CAT-Q and the behavioural maintenance factors of social anxiety in autistic adolescents. These behavioural maintenance factors were considered to be a form of ‘impression management’, the processes by which people try to control how others perceive them (Leary & Kowalski, 1990). Lei and colleagues suggested that camouflaging/masking may be a perpetuating factor in maintaining social anxiety in autistic young people.

In the third factor analysis exploring construct overlap between CAT-Q items and autistic social traits (Figure 1(c); Table 6), some CAT-Q Assimilation items loaded together with social BAPQ items in three of the four factors derived. Although most of the CAT-Q Masking and Compensation items were largely separable from social autistic trait items in terms of their factor loadings, there was some overlap between CAT-Q Assimilation and autistic social traits. Specifically, the CAT-Q Assimilation items were split between a factor mostly containing items from the CAT-Q Masking subscale and a factor with BAPQ Aloof subscale items, suggesting that these two subscales likely have some construct overlap. This finding was further corroborated by the positive correlation between the CAT-Q Assimilation and the BAPQ Aloof subscale found in both our study (*r* = 0.65) and in Hull et al. (2019; *r* = 0.63). In particular, two Assimilation items (Item 4 ‘I have to force myself to interact with people when I am in social situations’ and Item 5 ‘when in social situations, I try to find ways to avoid interacting with others’) loaded exclusively on a factor otherwise comprising only of BAPQ Aloof items. Given Hull et al.’s (2019) description of the Assimilation subscale as ‘attempts to blend in to social situations in which the individual is uncomfortable, without letting others see this discomfort’ (p. 10), it appears these Assimilation items were intended to capture social discomfort or distress when trying to blend in socially (Hull et al., 2019), in contrast to the BAPQ Aloof items which capture reduced motivation or disinterest in social interactions (Hurley et al., 2007). It is possible that these two CAT-Q Assimilation items may require refinement so they do not also inadvertently describe social motivation.

In addition, although most CAT-Q items differentiated well from the social anxiety items in the factor analysis, there may be some overlap between the CAT-Q Assimilation items and three social interaction anxiety items (‘I find it difficult mixing comfortably with the people I work with’, ‘I tense up if I meet an acquaintance on the street’ and ‘I have difficulty talking with other people’), as these loaded together on one factor. Notably, all three social interaction anxiety items were from the SIAS and all described some discomfort in social interactions, consistent with the CAT-Q Assimilation subscale items. Together with the overlap between CAT-Q Assimilation and BAPQ Aloof autistic social traits discussed earlier, these findings may indicate that the CAT-Q Assimilation items in particular may be less distinct than the other CAT-Q subscale items and may overlap to some extent with social interaction anxiety and autistic social traits. Interestingly, the CAT-Q Compensation and Masking subscales were anticipated factors in the original Hull et al., (2019) factor analyses, but the Assimilation factor was unexpected. This factor was interpreted by Hull and colleagues to reflect efforts to ‘fit in’ and hide discomfort in social situations, an aspect of camouflaging that was conceded by the authors to be less explored in previous literature. Thus, interpretation of the latent construct assumed to underlie the CAT-Q Assimilation items may be more difficult compared to the Masking and Compensation subscale items. Another possibility may be that the CAT-Q Assimilation items, which mostly describe the person’s emotional experiences in relation to camouflaging, load together with social anxiety items because they are more similar to the emotionally and cognitively focused items of the SIAS/SPS social anxiety measure. Based on our findings, the CAT-Q Assimilation subscale may need to be interpreted with more caution than the other CAT-Q subscales and may require further refinement or development. Overall, however, the construct validity of the CAT-Q as a measure of camouflaging is supported by our findings.

### Strengths, limitations and future directions

The present study is, to the best of our knowledge, the first study specifically exploring the construct overlap of the CAT-Q with other measures. A more comprehensive and detailed approach was taken to explore the construct validity of the CAT-Q via factor analysis than had previously been explored.

In terms of limitations, no autistic adults with verbal and/or intellectual impairments were included in our group of participants, and thus the results cannot be generalised to autistic people with these profiles and characteristics. Our participants were also predominantly White with a large percentage of females, so for greater confidence in generalisability, these analyses would need to be replicated with more diverse autistic samples, especially given that there may be important sociocultural influences in camouflaging that need to be considered and investigated further (e.g. Oshima et al., 2024; Tamura et al., 2024; Zhuang et al., 2024).

Our analyses should be replicated and extended, and other approaches to assessing construct validity should be explored. For example, as suggested by Williams (2022), quantitative semantic analysis, which can detect semantic overlap between two potentially related scales (Rosenbusch et al., 2020) offers another approach to exploring construct validity. In addition, in our study, most of the autistic participants reported high social anxiety, with more than 90% exceeding the general population measure’s cut-off. While not uncommon, our reported rates are higher than those found in a meta-analysis based on self-report measures (51%; Hollocks et al., 2019). It is possible that the brief scales used may partly explain these high rates in this study. Another possibility is that autistic participants experiencing anxiety in social interactions may have been more likely to participate in a study about camouflaging and its psychosocial correlates and consequences. Although social anxiety and camouflaging are commonly interrelated, autistic people with lower social anxiety also report camouflaging (Cage & Troxell-Whitman, 2019). For greater generalisability, future studies with larger samples could explore construct overlap separately for autistic individuals with higher versus lower social anxiety (or with and without diagnoses of social anxiety).

The results of the present study lend more confidence to using the CAT-Q for research, while at the same time suggest that the CAT-Q should be further developed and evaluated. The relationship between camouflaging, social anxiety and impression management needs to be explored and disentangled further in autistic people as well as in other diagnostic and social groups, especially the CAT-Q Assimilation subscale items (see Ai et al., 2022). As the CAT-Q items were related with, but largely factorially separable from, the social anxiety and the autistic social traits’ items, the CAT-Q could be useful in clinical practice as an additional tool aiding the exploration of psychosocial and mental health issues affecting autistic people: when issues relating to possible camouflaging are discussed in assessment or therapy, inviting autistic adults to complete the CAT-Q and to then discuss and share the extent to which they camouflage, and their person-specific reasons and consequences of camouflaging may provide additional helpful insights into some autistic people’s psychosocial experiences which can improve clinicians’ understanding and formulation of their mental health and wellbeing difficulties.

## Summary and conclusion

This study examined the CAT-Q’s construct validity by exploring its relationship to, and separability from, measures of related constructs (social anxiety, FNE and autistic social traits). We found that the CAT-Q items were related to, but also largely factorially separable from, social anxiety, FNE and social autistic trait items, strengthening confidence in the construct validity of the CAT-Q in measuring camouflaging behaviours. The CAT-Q Assimilation subscale items, however, showed some overlap with social anxiety and autistic social traits and may therefore require attention, more cautious interpretation and potentially refinement. It is important to continue the iterative improvement of the CAT-Q and other camouflaging measures to strengthen how we measure camouflaging, so that research can continue to answer important questions regarding autistic people’s experiences.

## Notes

1. The *Diagnostic and Statistical Manual of Mental Disorders* (5th ed., text rev.; DSM-5-TR; American Psychiatric Association, 2022) and much previous research have adopted medical and deficit focused language and perspectives to autism. In response to the limitations of this deficit-based understanding of autism (Pellicano & den Houting, 2022), and to align with the language preferences of the autistic community (Bottema-Beutel et al., 2021), in this study we use ‘autism’ instead of ‘ASD’, ‘differences’ and/or ‘difficulties’ instead of symptoms or deficits, and so on.

2. Other terms related, or synonymous, to camouflaging include masking, passing, accommodation, compensation and adaptive morphing (Lawson, 2020; Libsack et al., 2021).

3. Self-identified autistic individuals were included in line with previous studies and the acceptance of self-identification within the autistic community, and in order to increase representation of those facing barriers to professional diagnosis or not professionally diagnosed due to camouflaging (Bradley et al., 2021; Cage et al., 2018; Libsack et al., 2021; Livingston et al., 2019).
